# Gene Expression Responses Linked to Reproduction Effect Concentrations (EC_10,20,50,90_) of Dimethoate, Atrazine and Carbendazim, in *Enchytraeus albidus*

**DOI:** 10.1371/journal.pone.0036068

**Published:** 2012-04-27

**Authors:** Sara C. Novais, Wim De Coen, Mónica J. B. Amorim

**Affiliations:** 1 Department of Biology & CESAM, University of Aveiro, Aveiro, Portugal; 2 Department of Biology - E.B.T., University of Antwerp, Antwerp, Belgium; Institut Jacques Monod, France

## Abstract

**Background:**

Molecular mechanisms of response to pesticides are scarce and information on such responses from soil invertebrates is almost inexistent. *Enchytraeus albidus* (Oligochaeta) is a standard soil ecotoxicology model species for which effects of many pesticides are known on survival, reproduction and avoidance behaviour. With the recent microarray development additional information can be retrieved on the molecular effects.

**Methodology/Principal Findings:**

Experiments were performed to investigate the transcription responses of *E. albidus* when exposed to three pesticides – dimethoate (insecticide), atrazine (herbicide) and carbendazim (fungicide) – in a range of concentrations that inhibited reproduction by 10%, 20%, 50% and 90% (EC_10_, EC_20_, EC_50_ and EC_90_, respectively). The goal of this study was to further identify key biological processes affected by each compound and if dose-related. All three pesticides significantly affected biological processes like translation, regulation of the cell cycle or general response to stress. Intracellular signalling and microtubule-based movement were affected by dimethoate and carbendazim whereas atrazine affected lipid and steroid metabolism (also by dimethoate) or carbohydrate metabolism (also by carbendazim). Response to DNA damage/DNA repair was exclusively affected by carbendazim.

**Conclusions:**

Changes in gene expression were significantly altered after 2 days of exposure in a dose-related manner. The mechanisms of response were comparable with the ones for mammals, suggesting across species conserved modes of action. The present results indicate the potential of using gene expression in risk assessment and the advantage as early markers.

## Introduction

Pesticides are a common source of pollution, being present at a large scale in many European soils. Pesticides are designed to affect a certain class of organisms (e.g. insecticides, herbicides, fungicides) but they also affect non-target organisms. The modes on how these pesticides affect non-target species are even less known and to understand its risks still constitutes a challenge.

Dimethoate is one of the most used insecticides in agricultural fields and it is known as a cholinesterase inhibitor acting at the cholinergic synapses of insects [1]. This ability to inhibit cholinesterases has been demonstrated for other groups of organisms like freshwater shrimps [2], chironomids [3], fish [4] or earthworms [5]. Besides these effects related to its mode of action in insects, dimethoate have been described to inhibit steroidogenesis in rats [6]. Atrazine is an herbicide, also widely applied, which has the function of inhibiting photosynthesis in photosystem II of plants. In frogs, fish and rats, atrazine has been described as a possible endocrine disruptor and as an immunotoxin [7]–[11]. Carbendazim is the predominant metabolite of the systemic broad spectrum fungicide benomyl, known for affecting the nucleus division by inhibiting microtubule assembly in fungi [12], [13]. The antimitotic action of carbendazim has also been described for mammals [14].

Toxicity mechanisms of these three pesticides on invertebrates, and in particular on soil invertebrates, are by far less known. *Enchytraeus albidus* (Oligochaeta) are an ecologically relevant invertebrate species and present in soils worldwide. They play a key role in the functioning of the soil ecosystem, being involved in the degradation of organic matter and improving the pore structure of the soil [15]. These organisms are often used as test species for soil toxicity testing [16], [17] and studies have reported effects of dimethoate, atrazine and carbendazim on its survival, reproduction and avoidance behaviour [18]–[22]. The mechanisms of such toxicity are still to be understood.

The development of a cDNA microarray for *E. albidus* [23], [24] provided a new tool to assess molecular mechanisms of pesticide toxicity. This microarray was enhanced (Agilent oligonucleotide microarray) with the development of two new cDNA libraries enriched with metal and pesticide responsive genes, and all the sequence information was made available in EnchyBASE (http://bioinformatics.ua.pt/enchybase/) [25].

The main goal of the present study was to investigate the early transcription responses of *E. albidus* to three pesticides with different modes of action: dimethoate, atrazine and carbendazim (as mentioned above). Ultimately, the objective is also to identify key biological processes affected that indicate mechanisms of toxic action for each pesticide. Gene expression studies of pesticides in invertebrates are still scarce and, to the authors' knowledge, this is the first transcriptomic study of dimethoate and carbendazim effects in invertebrates. Transcription effects of atrazine have been assessed in a few studies [26]–[28].

In order to further understand the underlying transcription responses for effects at higher levels of biological organization, organisms were exposed for 2 days to a range of concentrations with known effects on reproduction – EC_10_, EC_20_, EC_50_ and EC_90_ – causing 10, 20, 50 and 90% reduction in the number of juveniles, respectively.

## Methods

### Maintenance of test species

*Enchytraeus albidus* [29] were maintained in laboratory cultures under controlled conditions with a photoperiod 16∶8 h light∶dark and a temperature of 18°C. Animals were fed twice a week with finely ground and autoclaved rolled oats.

### Soil and spiking of the test substances

Spiking of all pesticides was performed in the natural standard soil LUFA 2.2 [pH (CaCl_2_) = 5.5; Organic Matter = 4.4%; texture = 6% clay, 17% silt, 77% sand]. Three pesticides were used in this study: the insecticide dimethoate [Sigma-Aldrich (Riedel-de Haën), 99.8%], the herbicide atrazine (Sigma-Aldrich, 97.4%) and the fungicide carbendazim (Sigma-Aldrich, 97%). Dimethoate was spiked into the pre-moistened soil as aqueous solution, each test concentration into the whole batch of soil for all replicates. For the non-water soluble chemicals (atrazine and carbendazim) acetone was used as solvent, being this solution homogeneously mixed with the soil and left to evaporate overnight. A solvent control was also performed – control soil spiked with the same amount of acetone. The soils were then moistened to 50% of the soil maximum water holding capacity (WHC).

Four concentrations of each pesticide were tested, based on previous results on reproduction effects [19]. The concentrations used were in the range of the 95% confidence intervals for the Effect Concentrations (EC) that caused 10, 20, 50 and 90% effects on reproduction (EC_10_, EC_20_, EC_50_ and EC_90_) and are presented in Table 1.

**Table 1:** Concentrations of dimethoate, atrazine and carbendazim used for exposures in *Enchytraeus albidus*.

	EC_10_ (mg/kg)	EC_20_ (mg/kg)	EC_50_ (mg/kg)	EC_90_ (mg/kg)
*Insecticide*				
**Dimethoate**	0.1	0.5	2.0	25
*Herbicide*				
**Atrazine**	0.2	0.5	3.0	50
*Fungicide*				
**Carbendazim**	0.05	0.1	0.5	3.0

Concentrations of exposure were based on the effect concentrations on reproduction [19].

EC_10_ = Concentration that induces 10% reduction in the number of juveniles; EC_20_ = Concentration that induces 20% reduction in the number of juveniles; EC_50_ = Concentration that induces 50% reduction in the number of juveniles; EC_90_ = Concentration that induces 90% reduction in the number of juveniles.

### Exposure conditions

Ten adult worms with well developed clitellum were introduced in the test vessels, each containing 25 g of moist soil. The worms were exposed for 2 days at 20°C and a 16∶8 h photoperiod. Three replicates per treatment were performed. In the end of the exposure, animals of each replicate were carefully removed, rinsed in deionised water, stored in RNA later (Ambion, USA) containing criotubes and frozen in liquid Nitrogen. All samples were stored at -80°C till further analysis.

### RNA extraction, labelling and hybridizations

Three biological replicates of each pesticide exposure condition and also of both control and solvent control conditions were used. The following procedures for the microarray experiment were the same as described in Novais et al. [30]. Briefly, isolation of total RNA from each replicate was performed with the Trizol extraction method (Invitrogen, Belgium) followed by a DNAse treatment (Fermentas, Germany) and further purification steps consisting of phenol/chloroform extractions. The quantity and purity of the isolated RNA were measured with nanodrop (NanoDrop ND-1000 Spectrophotometer) and its integrity was checked on a denaturing formaldehyde agarose gel electrophoresis.

A single-colour design was used starting from 500 ng of total RNA which was amplified and labelled with the Agilent Low Input Quick Amp Labelling Kit (Agilent Technologies, Palo Alto, CA, USA). Positive controls were added with the Agilent one-colour RNA Spike-In Kit (Agilent Technologies, Palo Alto, CA, USA). Purification of the amplified and labelled cRNA was performed with the RNeasy columns (Qiagen, Valencia, CA, USA).

The cRNA samples were hybridized on Custom Gene Expression 60-mer oligonucleotide Agilent Microarrays (8×15 k format) developed for this species [25], [30]. Hybridization was performed using the Agilent Gene Expression Hybridization Kit (Agilent Technologies, Palo Alto, CA, USA) and each biological replicate was individually hybridized on one array. After the 17 h hybridization at 65°C with a rotation of 10 rpm, microarrays were washed using Agilent Gene Expression Wash Buffer Kit (Agilent Technologies, Palo Alto, CA, USA) and scanned with the Agilent DNA microarray scanner G2505B (Agilent Technologies). A total of 42 hybridizations resulted from the present microarray experimental design (four concentrations – EC_10_, EC_20_, EC_50_ and EC_90_ – of three pesticides – dimethoate, atrazine and carbendazim – in triplicate, plus three replicates of control and solvent control conditions).

### Analysis of microarray data

Fluorescence intensity data was obtained with Feature Extraction (10.5.1.1) Software (Agilent Technologies). Quality control was done by inspecting the reports on the Agilent Spike-in control probes and by making box plots of each array. Processing of the data and statistical analysis were performed using BRB Array Tools version 4.1 Stable Release (http://linus.nci.nih.gov/BRB-ArrayTools.html). After background subtraction, the replicated spots within each array were averaged and the intensities were log_2_ transformed. Data was then normalized using median array as reference. Raw and processed data are available from Gene Expression Omnibus (GEO) at the NCBI website (platform: GPL14928; series: GSE33945).

Given that two of the pesticides were dissolved in acetone (atrazine and carbendazim), comparisons of those exposure treatments were made against the solvent control, whereas dimethoate treatments were compared to the water control. Statistical class comparison was performed between those different groups of arrays using two-sample t-test with 95% confidence level for the assessment of differentially expressed genes and the log_2_ ratios of expression between those classes were calculated and used for further analysis.

Annotation of the differentially expressed genes (p<0.05) was performed based on their similarity to sequences in the National Centre for Biotechnology Information (NCBI) database as determined by the Basic Local Alignment Search Tool (BLAST) [31]. The sequences were submitted to Blast2GO [32] being compared with peptide sequence databases using BLASTX analysis (with *e*-value<10^−5^). GO term enrichment analysis [33] was performed for the differentially expressed genes using the same Blast2GO software. Clustering and principal component analyses (PCA) were performed using MultiExperiment Viewer (MeV, TIGR).

### Quantitative Real-Time PCR confirmations

Total RNA (1 µg) from all control and pesticide exposed samples was converted into cDNA through a reverse transcription reaction using the SuperScript First-Strand Synthesis System for RT-PCR (Invitrogen). Amplification was performed using Platinum SYBR Green qPCR SuperMix-UDG (Invitrogen) on the 7500 Real-Time PCR System (Applied Biosystems). Primer sets were designed for seven target genes (EAC00488, EAC00716, EAC00387, EAC00992, EAC00791, EAC00265, EAC00181; see Table S1) and one endogenous control gene (myosin alkali light chain 1 – EAC00302) with the software Oligo ExplorerTM (version 1.1.0). Efficiency and specificity of each primer was determined by observing the obtained standard and melting curves, respectively, for all primer sets. Primer sequences can be found in EnchyBASE – http://bioinformatics.ua.pt/enchybase/ [25].

Quantitative real-time PCR (qPCR) was performed with three biological replicates of each condition (the same used for the microarray experiment), applied in triplicate on a 96-well optical plate (GeneAmp®, Applied Biosystems). Reaction conditions consisted of one initial cycle at 50°C for 2 min, followed by a denaturation step at 95°C for 2 min, 40 cycles at 95°C for 32 sec and 1 cycle at 60°C for 1 min. Finally, a dissociation step was made consisting of 15 sec at 95°C, 1 min at 60°C, and 15 sec at 95°C.

A mean normalized expression value was calculated from the obtained Ct values of the test genes with Relative Expression Software Tool (REST-MSC).

## Results

Transcription dose-responses were determined for the three pesticides from different classes: the insecticide dimethoate, the herbicide atrazine and the fungicide carbendazim.

Analysis of the statistical class comparison results (two sample t-tests, p<0.05) between control and each of the dimethoate treatments resulted in a total of 317 significant differentially expressed genes. The comparison between solvent control and atrazine or carbendazim conditions resulted in 161 and 334 significant differentially expressed genes, respectively. From these transcripts, 135 (43%), 51 (32%) and 127 (38%) for dimethoate, atrazine and carbendazim exposures respectively, match known proteins in public databases. The complete list of affected genes with significant blast homologies, is given in Table S2.

**Figure 1:**
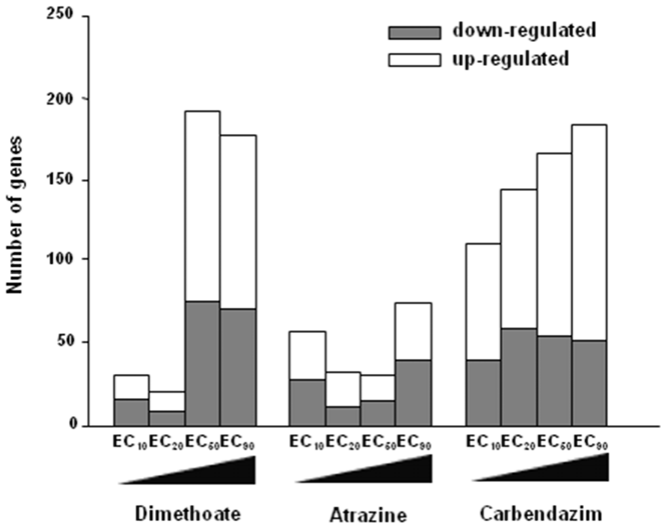
Number of differentially expressed genes after dimethoate, atrazine and carbendazim exposures. Number of significant up- and down-regulated genes (two sample *t*-tests, p<0.05) in *Enchytraeus albidus* after exposure to dimethoate, atrazine and carbendazim in four different concentrations (reproduction EC_10_, EC_20_, EC_50_ and EC_90_). Values refer to the comparison with the respective control.

The total number of over and under expressed transcripts in each of the pesticide conditions is represented in Figure 1.

In general, higher concentrations of the tested pesticides affected more transcripts than lower ones. Atrazine affected the lowest number of transcripts in total (Figure 1). In exposure to carbendazim it was possible to observe dose-response relation, increasing the number of differentially expressed genes with increasing concentrations. There was also a tendency for higher up-regulation with increasing concentrations (Figure 1).

**Figure 2:**
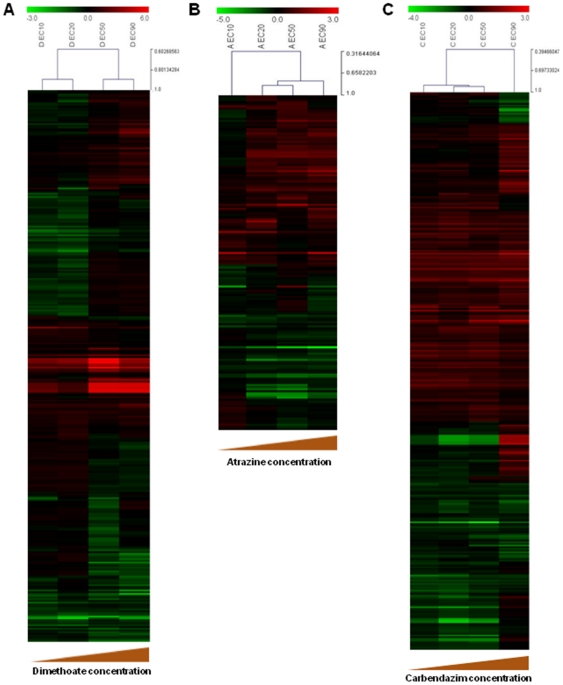
Heat map and Clustering analysis of dimethoate, atrazine and carbendazim samples. Heat map and hierarchical clustering of samples (Pearson's uncentered) based on the differentially expressed genes (two sample *t*-test, p<0.05) in *Enchytraeus albidus* when comparing: **A –** dimethoate exposure treatments against control; **B –** atrazine exposure treatments against solvent control; **C –** carbendazim exposure treatments against solvent control.

The effect of dosage on the transcriptional profiles becomes clearer from the clustering analysis of samples from each pesticide treatment (Figure 2).

The gene expression was concentration dependent and showing a distinct chemical-related pattern. In dimethoate exposures (Figure 2 – A), the two lower and two higher concentrations were grouped separately, whereas for atrazine and carbendazim (Figure 2 – B, C) the concentrations that cause 20% and 50% effect on reproduction were more closely related.

**Figure 3:**
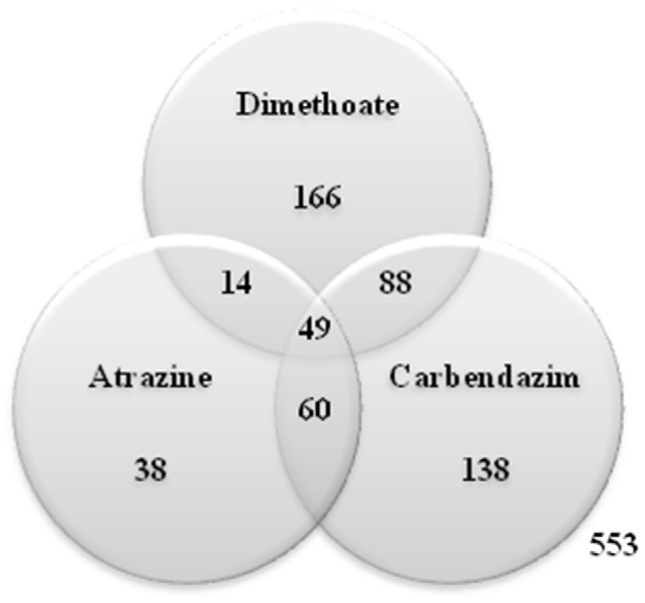
Venn diagram representing the number of differentially expressed genes shared by the three pesticides.

Expression profiles show distinct patterns for each pesticide (Figure 2), suggesting that responses occur through different molecular pathways. These different responses are depicted by the different directions in which the same genes are affected (up- or down-regulation) and by the uniquely affected transcripts in each pesticide exposure. The number of common and uniquely affected transcripts is represented in the Venn diagram of Figure 3.

The number of overlapping genes (Figure 3), as an indicative of a common response, is higher between carbendazim and dimethoate. Atrazine seems to induce a more similar response to carbendazim than to dimethoate and, in fact, only 38 transcripts were exclusively affected by this herbicide. Overall, 49 transcripts are affected by all compounds which may represent general stress responses to stress. From the 49 genes, 11 have known homologies and code for e.g. heat shock protein 90, lombricine kinase, neutral and basic amino acid transport protein or integrin-linked kinase-associated serine threonine phosphatase 2c (ILKAP).

The seven different sets of differentially expressed genes, as presented in the Venn diagram (Figure 3), correspond to 3 lists of uniquely affected transcripts by each pesticide and 4 lists of transcripts shared by two or three of these compounds. Those lists were used to perform an improved gene set enrichment analysis of GO terms [33] and evaluate the biological functions significantly affected in each case (Table S3). All differentially expressed genes, with significant blast homologies, present in each of the seven lists used for this analysis can be found in Table S4.

GO enrichment analysis determined several biological processes (GO terms) as significantly more abundant in the differentially expressed gene lists than would be expected by chance (Table S3). Based on this analysis, all three pesticides significantly affected (p<0.05) biological processes like translation, regulation of the cell cycle or response to stress with chaperone proteins. Intracellular signalling and microtubule-based movement were biological processes found to be significantly affected after exposure to dimethoate and carbendazim. Atrazine affected other biological processes like lipid, steroid and RNA metabolisms (also affected by dimethoate) and carbohydrate metabolism (also affected by carbendazim). Response to DNA damage/DNA repair was a process only found to be significantly affected after carbendazim exposures. All significant differential transcripts within each GO term are given in Table S5.

**Figure 4:**
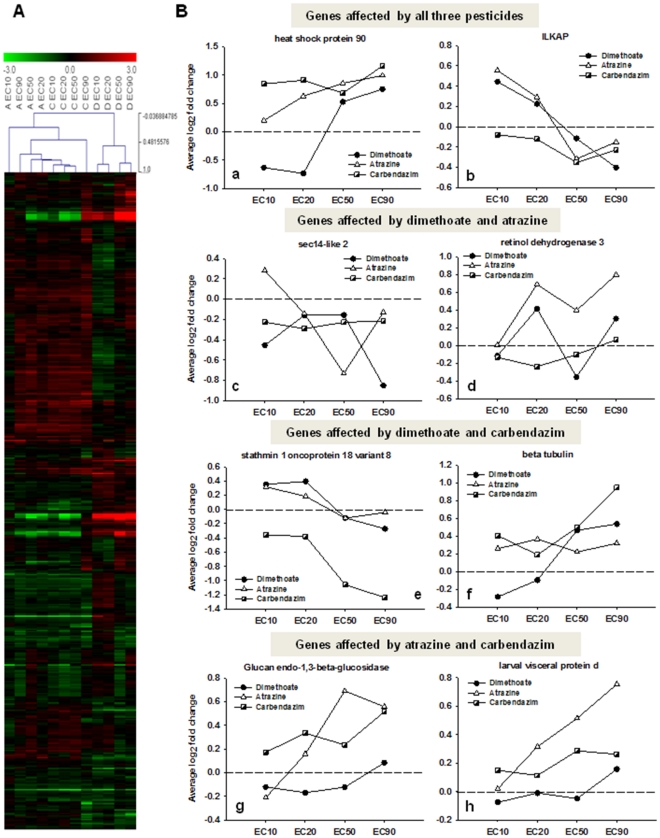
Clustering analysis of all samples and expression of transcripts affected by more than one pesticide. **A -** Heat map and hierarchical clustering of samples (Pearson's uncentered) based on the differentially expressed genes (two sample *t*-test, p<0.05) in *Enchytraeus albidus* exposed to four concentrations of dimethoate, atrazine and carbendazim (reproduction EC_10_, EC_20_, EC_50_ and EC_90_). **B –** Expression behavior of some transcripts significantly affected by two or by the three pesticides (dimethoate, atrazine and carbendazim), across a range of concentrations: **a** – heat shock protein 90; **b** – integrin-linked kinase-associated serine threonine phosphatase 2c (ILKAP); **c** – sec 14-like 2; **d** – retinol dehydrogenase 3; **e** – stathmin 1 oncoprotein 18; **f** – beta tubulin; **g** – glucan endo-1,3-beta-glucosidase; **h** – larval visceral protein d.

Although several genes, and consequently biological processes, were affected by two or even by the three pesticides tested, some of these transcript expressions were negatively correlated, which can be seen in the heat map with the whole gene expression profiles (Figure 4 – A). From this clustering analysis it is possible to observe that effects of atrazine and carbendazim were more closely related than effects of dimethoate. The behaviour of gene expression change across the range of concentrations of each pesticide is represented in Figure 4 for some of the significant differentially expressed transcripts involved in the biological processes mentioned above (Figure 4 – B).

As it can be seen for some of the differentially expressed genes in Figure 4B, these are affected by more than one pesticide, but the expression pattern differs with increasing exposure concentrations (e.g. sec 14-like 2, larval visceral protein d). Moreover, in other cases genes have the same dose-effect relations but the intensities of expression are different (e.g. retinol dehydrogenase 3) or even opposite (e.g. stathmin 1 oncoprotein 18, at EC_10_ and EC_20_).

To validate the microarray results, seven genes involved in the significantly affected biological functions were selected for qPCR quantification. For each pesticide exposure, the expression of 4 different genes was confirmed by qPCR, in all four concentrations, using myosin alkali light chain 1 (EAC00302) as housekeeping (Table S1).

**Figure 5:**
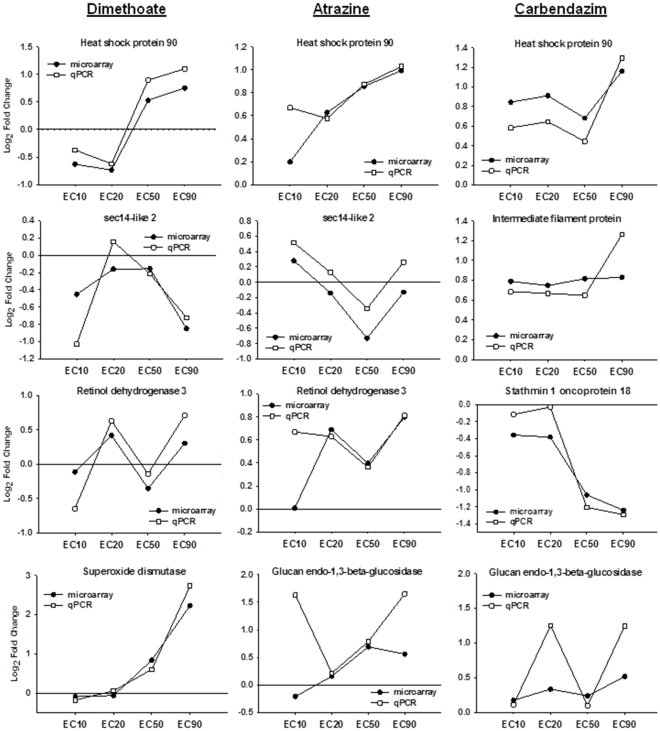
Comparison between microarray and qPCR gene expression analysis. Representation of average log_2_ fold change expression values obtained for the genes coding for heat shock protein 90, sec-14 like 2, intermediate filament protein, retinol dehydrogenase 3, stathmin 1 oncoprotein 18, superoxide dismutase and glucan endo-1,3-beta-glucosidase with microarrays (black circles) and qPCR (white squares) after exposure of *Enchytraeus albidus* to concentrations of dimethoate, atrazine or carbendazim causing 10%, 20%, 50% and 90% reduction in reproduction (EC10, EC20, EC50 and EC90, respectively).

By comparing gene expression results of both platforms, microarray and qPCR (Figure 5), it is possible to observe that responses were very similar, following the same dose-response patterns. Responses had minute variations in fold change magnitudes and their direction (up- or down-regulation) was coherent, which confirms results. A significant correlation of 0.845 (Pearson's correlation, p = 4.503E-014, n = 48) was obtained between the two platforms (Figure S1).

## Discussion

Overall, and considering all the concentrations, the herbicide atrazine was the compound that induced less gene expression changes. This is not particularly surprising given that this is a compound designed to affect mainly plant organisms. Additionally, studies in fish and human cell lines with atrazine did not reveal significant changes in gene expression [34], [35].

In general there was an increase in the number of affected transcripts with increasing concentrations. This tendency was also observed in a study with *E. albidus* exposed to Cd and Zn [30]. On the contrary, this was not the pattern when Cu was tested in this species [36] or phenanthrene in the collembolan *Folsomia candida* [37]. Although the same effect concentrations on reproduction were tested for all pesticides, the gene expression correlation between concentrations was different depending on the pesticide (Figure 2). These results indicate different mechanisms underlying reductions on the reproduction rates. Gene results in terms of number seem to follow the steepness of the reproduction dose-responses curves as observed for the different chemicals [19] but it is difficult to fully overview under the present test design.

As shown in the Venn diagram (Figure 3), certain gene responses were shared by all three pesticide exposures or by two of them. Due to the fact that *E. albidus* does not have a sequenced genome, many of the significant transcripts have no similarity to known proteins. Hopefully, with future sequencing efforts and with the growing genomic data on invertebrate species, more transcripts will be annotated.

Dimethoate affected transcripts related with sarcomere organization, maintenance of cell polarity and response to calcium ion. These transcripts code for several actin, calponin, toponin and sarcoplasmic calcium binding proteins which were significantly affected mainly at the EC_10_ and EC_20_ dimethoate concentrations. The clear separation in expression between the lowest and highest concentrations were also observed for several transcripts related with the electron transport system, from complex I (NADH dehydrogenase), complex III (cytochrome b) and complex IV (cytochrome c oxidase) and ATP synthase from which ultimately the energy is produced. All of these transcripts were up-regulated at lower doses and then down-regulated at higher doses, suggesting an inhibition of the electron transport chain and consequent ATP production with increasing concentrations. Dysfunction of the mitochondrial respiratory chain has been associated with increased peroxide and hydrogen peroxide production in cells [38] and consequent alterations in the activity of antioxidant enzymes [39]. Interestingly, dimethoate induced the transcript coding for the antioxidant enzyme superoxide dismutase (SOD) in a concentration related manner (which was qPCR confirmed, Figure 5). These transcripts with opposite expression between the lower and higher concentrations of dimethoate play an important role in the way dimethoate clustered as opposed to the way atrazine and carbendazim samples clustered (Figure 4 – A). Overall, responses to the EC_10_ and EC_20_ of dimethoate [discussed above] are opposite from those of atrazine and carbendazim, being less distinct at EC_50_ and EC_90_.

Information on mechanisms of dimethoate toxicity in other organisms is very limited and what is known is that this compound has the ability to 1) inhibit acetylcholinesterase activity in several organisms [3], [5], [40] and to 2) have an influence on the metabolic pathways controlled by steroid hormones in rats [6], [41]. Inhibition of acetylcholinesterase activity could not be assessed from the present transcriptomic analysis since this particular transcript is not present in the library for this species. Additional work by the authors [42] confirmed this effect, as cholinesterases activity was inhibited after 8 days of exposure. As for the second known mechanism, there are evidences in the present study pointing to an inhibition of steroidogenesis. The under-expression of sec14-like 2 transcript (Figure 4B – c), involved in the positive regulation of cholesterol biosynthesis, suggests that less cholesterol will be synthesized and consequently, less steroids will be generated. Inhibition of steroidogenesis, along with the over expression of retinol dehydrogenase involved in the metabolism of vitamin A (Figure 4B – d), were common mechanisms between dimethoate and atrazine toxicity. Expression of the mentioned transcripts involved in these mechanisms was further confirmed by qPCR (Figure 5). The role of steroids on this particular species is not known and the association of steroids with their endocrine physiology has not yet been shown. However, in recent years more emphasis has been given to the study of invertebrate endocrine system showing that many steroid metabolic pathways are common to the ones of vertebrates and that some of the sex steroids have conserved functions in invertebrates reproduction as well [43], [44]. Further studies are required but our results might indicate a possible mechanism of endocrine disruption in *E. albidus*, where steroid and retinol metabolisms were disturbed, producing imbalanced levels of reproduction hormones.

From the uniquely affected transcripts after atrazine exposure, a gene coding for a histone was significantly up-regulated at the EC_10_. This protein is involved in biological processes related with cell adhesion or the regulation of cell shape and maintenance of DNA integrity. The functions of histones have also been linked to positive regulation of growth rate and larval development, and its enhancement was reported in a study where *Caenorhabditis elegans* was exposed to atrazine [45]. Some studies with atrazine have also reported disruption on the mitochondrial electron flow. Owen et al. [28] observed significant up-regulation of several transcripts coding for the oxidative phosphorylation pathway in *Lumbricus rubellus*. The proteomic approach used by Thornton et al. [46] in *Drosophila* m*elanogaster* exposed organisms also showed significant changes on the mitochondrial protein expressions. In our microarray results, transcripts coding for proteins of the electron transport system were mainly up-regulated after the EC_20_, confirming the assumption that atrazine affects the normal mitochondrial functioning. Along with carbendazim, atrazine also seems to affect the carbohydrate metabolism by enhancing gluconeogenesis. Both glucan endo-1,3-beta-glucosidase (Figure 4B – g) and larval visceral protein d (probable maltase, Figure 4B – h) transcripts were up-regulated after carbendazim and atrazine exposures, with validated expression levels by qPCR for the first mentioned transcript (Figure 5). This tendency for increased glucose storage has also been described in a study by Zaya et al. [47] where gene expression coding for glycolysis in *Xenopus laevis* suggested inhibition of this energetic process.

Carbendazim was the only pesticide to induce transcripts encoding for intermediate filament proteins which are involved in DNA ligation during DNA repair. Those transcripts are significantly up-regulated at all carbendazim concentrations (confirmed by qPCR – Figure 5) suggesting DNA damage and the indication of potential genotoxic effect of this pesticide, even at low concentrations. Effects of carbendazim on reproduction have been attributed to its well known function to interfere with the assembly of microtubules [12], rather than a mechanism involving endocrine disruption [48], [49]. Our results seem to be in good agreement with that hypothesis. Stathmin 1 oncoprotein 18 and several tubulin transcripts, that are differentially expressed by this compound, code for proteins directly involved in the regulation of cellular proliferation by assembling/disassembling microtubules. Stathmin 1 (oncoprotein 18) gene encodes for a cytoplasmic tubulin-binding phosphoprotein that acts to sequester tubulin and favour microtubule disassembly [50]. Disturbances in the normal expression of stathmin correlate with a decreased inactivation of tubulin, a constant microtubule and mitotic spindle assembly and a consequent incapacity to regulate cell cycle progression [51]. For this reason, disturbances in stathmin 1 expression have been associated with several types of cancer [50]–[52].

In the present study, the microtubule assembly/disassembly process seems to be affected not only by carbendazim but also by dimethoate. However, the gene response pattern is different for each pesticide (Figure 4B – e, f). Responses of stathmin 1 and tubulin in relation to the dose followed the same pattern in both pesticide exposures. Nevertheless, while tubulin was significantly up-regulated in both pesticides (except in the lower concentrations of dimethoate), stathmin 1 response was clearly different between the compounds. In dimethoate EC_10_ and EC_20_ the stathmin 1 expression was significantly up-regulated and only after the EC_50_ its inhibition started to occur, while in carbendazim its expression was severely inhibited in a dose-response related manner, a pattern further confirmed by qPCR analysis (Figure 5).

Other biological processes were affected by all three pesticides like the response to unfolded proteins with the up-regulation of several heat-shock proteins and chaperonins or the impairment of the normal regulation of cell cycle with the dose-dependent down-regulation of ILKAP gene (Figure 4B – a, b). Expression of one heat-shock protein 90 was also determined by qPCR in all pesticide conditions and confirmed the responses given by the microarray (Figure 5). Several transcripts related to protein catabolism were significantly over expressed in each pesticide exposure and, although they were not the same transcripts they all shared the same putative function.

Overall, the results show the importance of testing a range of concentrations to further understand and interpret the mechanisms of action. The fact that genes responded in a dose-related manner also suggests their usefulness in effect/risk assessment. Significant changes in gene expression can be observed after 2 days exposure, which can aid the interpretation of effects on reproduction, constituting a potential early indicator of phenotype effects.

This study provided novel information, contributing to unravelling the mechanisms of pesticide toxicity in invertebrates. Interestingly, some of the known mechanisms of action of these compounds in this soil invertebrate were comparable to the ones in mammals, suggesting across species conserved modes of action. This suggests that in the future *E. albidus* can be used as a model species within the 3R - refinement, reduction and replacement of animal testing, i.e. potentially useful to read across species.

Although studies have reported common mechanisms of action of tested compounds between invertebrates and those of mammals, the physiological consequences can be very different. This is an important topic to pursue because if further confirmed that mechanisms of action are common, it would mean that efforts could be shifted from unraveling the mechanisms of action to the step after, i.e., to translating the molecular mechanisms of action into physiological effects, hence predicting the toxicological effects for different organisms.

## Supporting Information

Figure S1 Correlation between gene expressions measured in *Enchytraeus albidus* using microarray analysis and qPCR. Each point represents the expression of a gene in one of the exposure conditions to dimethoate, atrazine or carbendazim.
(TIF)

Table S1 **Gene expression validation with quantitative Real Time PCR in** ***Enchytraeus albidus*** **when exposed to dimethoate, atrazine or carbendazim using reproduction EC10, EC20, EC50 and EC90.**
(XLSX)

Table S2 **Significant differentially expressed transcripts (two sample t-test, p<0.05) in** ***Enchytraeus albidus*** **when exposed to four concentrations of dimethoate, atrazine and carbendazim (reproduction EC10, EC20, EC50 and EC90).**
(XLSX)

Table S3 **Significant enriched GO terms (p<0.05) in** ***Enchytraeus albidus*** **in the following lists of differentially expressed genes:** 1) uniquely affected by dimethoate; 2) uniquely affected by atrazine; 3) uniquely affected by carbendazim; 4) affected by dimethoate and atrazine; 5) affected by dimethoate and carbendazim; 6) affected by atrazine and carbendazim; 7) affected by the three pesticides. Only the biological process results are given.
(DOCX)

Table S4 **Significant differentially expressed transcripts in** ***Enchytraeus albidus*** **with significant blast homologies, present in the following lists of differentially expressed genes:** 1) uniquely affected by dimethoate; 2) uniquely affected by atrazine; 3) uniquely affected by carbendazim; 4) affected by dimethoate and atrazine; 5) affected by dimethoate and carbendazim; 6) affected by atrazine and carbendazim; 7) affected by the three pesticides.
(XLSX)

Table S5 **All significant differential expressed genes of the significant (** ***p*** **<0.05) gene ontology (GO) terms in** ***Enchytraeus albidus*** **, in each of the following lists:** 1) uniquely affected by dimethoate; 2) uniquely affected by atrazine; 3) uniquely affected by carbendazim; 4) affected by dimethoate and atrazine; 5) affected by dimethoate and carbendazim; 6) affected by atrazine and carbendazim; 7) affected by the three pesticides.
(XLSX)
