# Supplementary figures and images for: Gene Expression Responses Linked to Reproduction Effect Concentrations (EC10,20,50,90) of Dimethoate, Atrazine and Carbendazim, in Enchytraeus albidus
Source: PLoS One. 2012 Apr 27;7(4):e36068. doi: 10.1371/journal.pone.0036068 (PMC3338630; doi:10.1371/journal.pone.0036068)

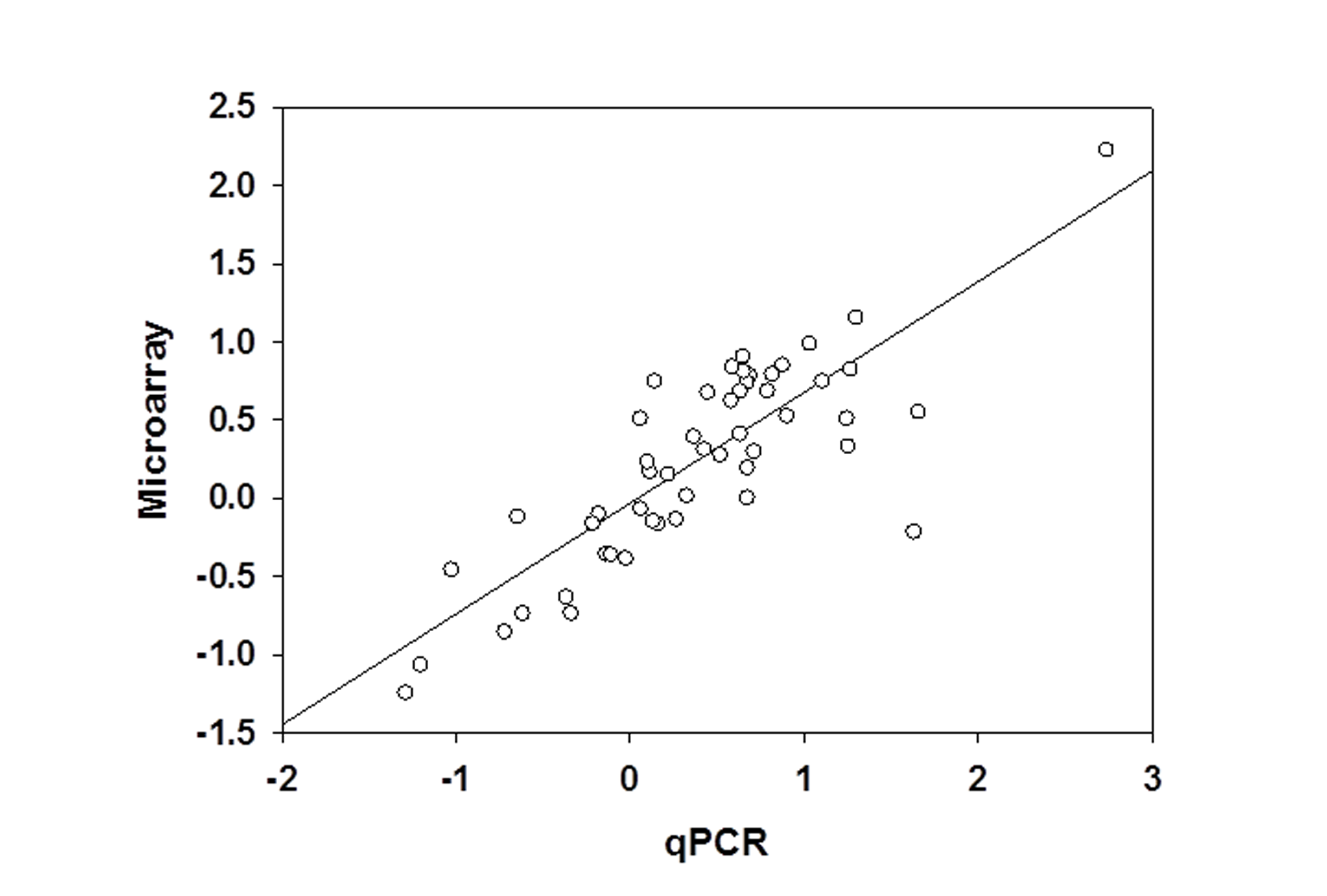

Supplement: Figure S1 — Correlation between gene expressions measured in Enchytraeus albidus using microarray analysis and qPCR. Each point represents the expression of a gene in one of the exposure conditions to dimethoate, atrazine or carbendazim. (TIF) [file pone.0036068.s001.tif]
