# Supplementary material for: Gene Expression Responses Linked to Reproduction Effect Concentrations (EC10,20,50,90) of Dimethoate, Atrazine and Carbendazim, in Enchytraeus albidus
Source: PLoS One. 2012 Apr 27;7(4):e36068. doi: 10.1371/journal.pone.0036068 (PMC3338630; doi:10.1371/journal.pone.0036068)
Supplement: Table S3 — Significant enriched GO terms (p<0.05) in Enchytraeus albidus in the following lists of differentially expressed genes: 1) uniquely affected by dimethoate; 2) uniquely affected by atrazine; 3) uniquely affected by carbendazim; 4) affected by dimethoate and atrazine; 5) affected by dimethoate and carbendazim; 6) affected by atrazine and carbendazim; 7) affected by the three pesticides. Only the biological process results are given. (DOCX) [file pone.0036068.s004.docx]

**Table S3.** Significant enriched GO terms (p < 0.05) in *Enchytraeus albidus* in the following lists of differentially expressed genes: 1) uniquely affected by dimethoate; 2) uniquely affected by atrazine; 3) uniquely affected by carbendazim; 4) affected by dimethoate and atrazine; 5) affected by dimethoate and carbendazim; 6) affected by atrazine and carbendazim; 7) affected by the three pesticides. Only the biological process results are given.

| **GO ID** | **GO term definition** | **Library annotated*^a^*** | **Significant*^b^*** | **p-Value** |
| --- | --- | --- | --- | --- |
| ***Genes uniquely affected by dimethoate*** | | | | |
| GO:0010035 | response to inorganic substance | 3 | 3 | 0.0039 |
| GO:0045214 | sarcomere organization | 4 | 3 | 0.0139 |
| GO:0050896 | response to stimulus | 23 | 8 | 0.0184 |
| GO:0032989 | cellular component morphogenesis | 11 | 5 | 0.0190 |
| GO:0051592 | response to calcium ion | 2 | 2 | 0.0252 |
| GO:0007163 | establishment or maintenance of cell polarity | 2 | 2 | 0.0252 |
| GO:0016070 | RNA metabolic process | 20 | 7 | 0.0266 |
| ***Genes uniquely affected by atrazine*** | | | | |
| GO:0051641 | cellular localization | 8 | 2 | 0.0199 |
| GO:0031532 | actin cytoskeleton reorganization | 1 | 1 | 0.0293 |
| GO:0007155 | cell adhesion | 1 | 1 | 0.0293 |
| GO:0032507 | maintenance of protein location in cell | 1 | 1 | 0.0293 |
| GO:0070972 | protein localization in endoplasmic reticulum | 1 | 1 | 0.0293 |
| GO:0008360 | regulation of cell shape | 1 | 1 | 0.0293 |
| GO:0006616 | SRP-dependent cotranslational protein targeting to membrane | 1 | 1 | 0.0293 |
| ***Genes uniquely affected by carbendazim*** | | | | |
| GO:0051716 | cellular response to stimulus | 4 | 3 | 0.0064 |
| GO:0048870 | cell motility | 4 | 3 | 0.0064 |
| GO:0006950 | response to stress | 13 | 5 | 0.0135 |
| GO:0006066 | alcohol metabolic process | 5 | 3 | 0.0145 |
| GO:0006259 | DNA metabolic process | 5 | 3 | 0.0145 |
| GO:0051103 | DNA ligation involved in DNA repair | 2 | 2 | 0.0148 |
| GO:0016477 | cell migration | 2 | 2 | 0.0148 |
| GO:0007243 | intracellular protein kinase cascade | 2 | 2 | 0.0148 |
| GO:0043408 | regulation of MAPKKK cascade | 2 | 2 | 0.0148 |
| GO:0023014 | signal transmission via phosphorylation event | 2 | 2 | 0.0148 |
| GO:0006412 | translation | 36 | 9 | 0.0206 |
| GO:0044267 | cellular protein metabolic process | 56 | 12 | 0.0254 |
| GO:0040011 | locomotion | 6 | 3 | 0.0266 |
| GO:0048511 | rhythmic process | 3 | 2 | 0.0408 |
| GO:0042274 | ribosomal small subunit biogenesis | 3 | 2 | 0.0408 |
| GO:0006281 | DNA repair | 3 | 2 | 0.0408 |
| GO:0006974 | response to DNA damage stimulus | 3 | 2 | 0.0408 |
| GO:0010033 | response to organic substance | 7 | 3 | 0.0425 |
| GO:0006414 | translational elongation | 12 | 4 | 0.0469 |
| ***Genes affected by dimethoate and atrazine*** | | | | |
| GO:0008202 | steroid metabolic process | 2 | 2 | 0.0004 |
| GO:0006629 | lipid metabolic process | 6 | 2 | 0.0057 |
| GO:0016070 | RNA metabolic process | 20 | 3 | 0.0062 |
| GO:0008203 | cholesterol metabolic process | 1 | 1 | 0.0213 |
| GO:0016101 | diterpenoid metabolic process | 1 | 1 | 0.0213 |
| GO:0006775 | fat-soluble vitamin metabolic process | 1 | 1 | 0.0213 |
| GO:0001523 | retinoid metabolic process | 1 | 1 | 0.0213 |
| GO:0006721 | terpenoid metabolic process | 1 | 1 | 0.0213 |
| GO:0006396 | RNA processing | 12 | 2 | 0.0237 |
| GO:0006412 | translation | 36 | 3 | 0.0326 |
| GO:0010467 | gene expression | 99 | 5 | 0.0328 |
| ***Genes affected by dimethoate and carbendazim*** | | | | |
| GO:0022607 | cellular component assembly | 20 | 5 | 0.0230 |
| GO:0007018 | microtubule-based movement | 4 | 3 | 0.0024 |
| GO:0043623 | cellular protein complex assembly | 7 | 4 | 0.0015 |
| GO:0023052 | signaling | 15 | 4 | 0.0340 |
| GO:0023034 | intracellular signaling pathway | 6 | 3 | 0.0104 |
| ***Genes affected by atrazine and carbendazim*** | | | | |
| GO:0007040 | lysosome organization | 1 | 1 | 0.0267 |
| GO:0006643 | membrane lipid metabolic process | 1 | 1 | 0.0267 |
| GO:0006665 | sphingolipid metabolic process | 1 | 1 | 0.0267 |
| GO:0007033 | vacuole organization | 1 | 1 | 0.0267 |
| GO:0005975 | carbohydrate metabolic process | 12 | 2 | 0.0367 |
| ***Genes affected by all 3 pesticides*** | | | | |
| GO:0044267 | cellular protein metabolic process | 56 | 5 | 0.0049 |
| GO:0006486 | protein amino acid glycosylation | 1 | 1 | 0.0240 |
| GO:0046209 | nitric oxide metabolic process | 1 | 1 | 0.0240 |
| GO:0042026 | protein refolding | 1 | 1 | 0.0240 |
| GO:0006470 | protein amino acid dephosphorylation | 1 | 1 | 0.0240 |
| GO:0007090 | regulation of S phase of mitotic cell cycle | 1 | 1 | 0.0240 |
| GO:0006464 | protein modification process | 12 | 2 | 0.0299 |
| GO:0034621 | cellular macromolecular complex subunit organization | 15 | 2 | 0.0457 |
| GO:0006986 | response to unfolded protein | 2 | 1 | 0.0475 |
| GO:0006449 | regulation of translational termination | 2 | 1 | 0.0475 |

*^a^* The number of annotated ESTs present in the library within the identified GO terms is indicated for comparison; *^b^* The number of GO term annotated differentially expressed ESTs in each of the seven gene lists.
